# MicroRNA-199a and -214 as potential therapeutic targets in pancreatic stellate cells in pancreatic tumor

**DOI:** 10.18632/oncotarget.7651

**Published:** 2016-02-24

**Authors:** Praneeth R. Kuninty, Linda Bojmar, Vegard Tjomsland, Marie Larsson, Gert Storm, Arne Östman, Per Sandström, Jai Prakash

**Affiliations:** ^1^ Department of Biomaterials, Science and Technology, Section: Targeted Therapeutics, MIRA Institute for Biomedical Technology and Technical Medicine, University of Twente, Twente, Netherlands; ^2^ Department of Clinical and Experimental Medicine, Linköping University, Linköping, Sweden; ^3^ Department of Pediatric Hematology/Oncology, Weill Cornell Medical College, New York, NY, USA; ^4^ Department of Hepato-pancreato-biliary Surgery, Institute of Clinical Medicine, University of Oslo, Oslo, Norway; ^5^ Department of Pharmaceutics, Utrecht University, Utrecht, Netherlands; ^6^ Department of Oncology-Pathology, Cancer Centre Karolinska, Karolinska Institutet, Karolinska, Sweden

**Keywords:** pancreatic cancer, pancreatic stellate cells, miRNA, stroma, cancer-associated fibroblasts

## Abstract

Pancreatic stellate cells (PSCs) are the key precursor cells for cancer-associated fibroblasts (CAFs) in pancreatic tumor stroma. In this study, we explored miRNA as therapeutic targets in tumor stroma and found miR-199a-3p and miR-214-3p induced in patient-derived pancreatic CAFs and TGF-β-activated human PSCs (hPSCs). Inhibition of miR-199a/-214 using hairpin inhibitors significantly inhibited TGFβ-induced differentiation markers (e.g. α-SMA, collagen, PDGFβR), migration and proliferation. Furthermore, heterospheroids of Panc-1 and hPSCs attained smaller size with hPSCs transfected with anti-miR-199a/-214 compared to control anti-miR. The conditioned medium obtained from TGFβ-activated hPSCs induced tumor cell growth and endothelial cell tube formation. Interestingly, these inductions were abrogated in hPSCs transfected with anti-miR-199a or miR-214. Moreover, IPA analyses revealed signaling pathways related to miR-199a (TP53, mTOR, Smad1) and miR-214 (PTEN, Bax, ING4). Taken together, this study reveals miR-199a-3p and miR-214-3p as major regulators of PSC activation and PSC-induced pro-tumoral effects, representing them as key therapeutic targets in pancreatic cancer.

## INTRODUCTION

Pancreatic ductal adenocarcinoma (PDA) is one of the most devastating cancers with a 5-year relative survival rate of less than 5% [1]. The survival rate is not improved over the past 30 years, despite the advent of many chemotherapies and radiotherapies [2, 3]. One of the main features of PDA is the desmoplastic reaction, characterized by the presence of abundant stroma that can occupy up to 90% of the whole tumor mass [4]. The role of pancreatic stroma has been contradictorily shown to possess anti-tumoral and pro-tumoral effects [5–7]. Olive et al. demonstrated that inhibition of hedgehog pathway using IPI-926 enhanced the efficacy of gemcitabine in a KrasLSL.G12D/+; p53R172H/+; PdxCretg/+ (KPC) mouse tumor model [7]. Meanwhile, clinical studies with a hedgehog inhibitor GDC-0449 failed to show the benefit in enhancing the anti-tumoral effect of gemcitabine [8]. Recently, depletion of tumor stroma either by deleting myofibroblasts genetically or by inhibiting hedgehog pathway (using an inhibitor or with Shh deficient tumors) accelerated the tumor growth with reduced survival [5, 6]. These studies provoked the debate on the tumor-supportive or tumor-inhibitory action of cancer-associated fibroblasts (CAFs) [9], suggesting a mixed population of CAFs [10]. Silencing of CAF pro-tumorigenic activities, instead of their depletion, might be the right direction to develop anti-stromal therapies to treat pancreatic cancer [9].

Pancreatic stellate cells (PSCs), the quiscent Vitamin A storing cells in pancreas, are the main precursor cells for CAFs [11]. During pancreatic injury or inflammation, PSCs are activated by pro-inflammatory cytokines and various growth factors and differentiate into alpha-smooth muscle actin (α-SMA)-expressing myofibroblasts [12–15]. The activated PSCs produce various growth factors, such as platelet-derived growth factor (PDGF), connective tissue growth factor (CTGF) and fibroblast growth factor-2 (FGF2) which in turn induce tumor cell proliferation, invasion, angiogenesis, and metastasis as well as confer development of resistance to chemotherapy [16–23]. Blockade of PSC activation might, therefore, be an interesting approach to inhibit their tumor–inducing actions.

MicroRNAs (miRNAs) are a class of small (approximately 22 nt) endogenous non-coding RNAs that inhibit the expression of hundreds of genes at the posttranscriptional level and thereby control cellular processes such as proliferation, differentiation, and apoptosis [24, 25]. Dysregulation of miRNA in stromal cells has received a huge attention for the potential therapeutic and diagnostic targets in cancer [26–29]. Several altered miRNAs such as miR-21, miR-143, and miR-210 have shown therapeutic significance on pancreatic tumor growth [30–32].

In the present study, we hypothesized that inhibition of miRNA, induced in activated PSCs, may inhibit the pro-tumoral effects of the activated PSCs. We selected miR-199a-3p and miR-214-3p as the main target for the investigation based on our miR array on stromal part isolated from colorectal tumors using laser capture dissection microscopy (Supplementary Figure 1) and the reported miRNA array in activated rat PSCs [33]. We first examined the expression levels of miR-199a and -214 in primary CAFs isolated from resected human pancreatic tumors and TGFβ-activated human hPSCs. Then, we investigated the effect of inhibition of either miR-199a or miR-214 on the differentiation, cell growth and migration of hPSCs and also on the hPSC-induced paracrine effects on human pancreatic tumor cells and endothelial cells *in vitro*. Lastly, we examined signaling pathways that are potentially responsible for the observed effects of the inhibition of these miRNAs.

## RESULTS

### Expression of miR-199a/-214 in pancreatic stroma, CAFs, and hPSCs

**Figure 1:**
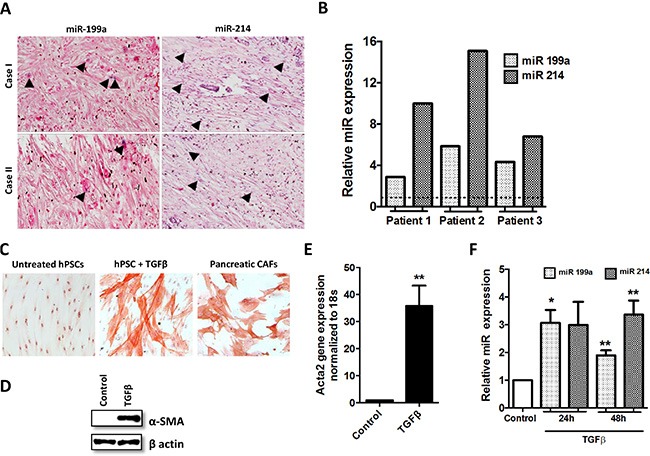
miRNA induction and phenotypic changes in TGF-β1 induced hPSCs differentiation (**A**) ISH detection of miR-199a/-214 in pancreatic cancer tissue; blue color staining shows the miR+ positive cells while the purple color represents the counterstaining. (**B**) Stem-loop RT-PCR validations confirmed differential expression of miRNAs in CAFs isolated from pancreatic head adenocarcinoma. (**C**) α-SMA staining (red color; magnification, 200×) and morphological changes following stimulation of hPSCs with TGF-β1 for 24 h in CAFs; (**D**, **E**) The expression of α-SMA was examined by western blots and real-time PCR after stimulating hPSCs with 5 ng/ml TGF-β1 for 24 h. (**F**) miRNA expression levels in the activated hPSCs after the treatment with 5 ng/ml TGF-β1 for 24 h and 48 h (**p < 0.01 vs control, *p < 0.05 vs control, *n* = 3, mean ± SEM).

To confirm that miR-199a and miR-214 are expressed in the stromal region of human pancreatic cancer, we first performed an *in-situ* hybridization (ISH) assay to detect the presence of miR-199a and miR-214 (Figure 1A). We found that both miR-199a and miR-214 were highly expressed (case I) and low expressed (case II) in the stroma of the human pancreatic tumors, which can be visualized as blue stained cells (see arrow heads). The expression levels of miR-199a and miR-214 were also confirmed in CAFs, which were isolated from three different patients (Figure 1B). In addition, we differentiated primary hPSCs with recombinant human TGF-β1, a well-known stimulant for stellate cells [34]. As shown in Figure 1C–1E, hPSCs were stretched with stress fibers and expressed high levels of α-SMA, a specific marker for myofibroblasts, after the treatment with TGF-β1. At last we compared the miRNA expression levels in non-activated and TGF-β activated hPSCs and found that both miR-199a and miR-214 were significantly induced in the activated hPSCs compared to that of non-activated hPSCs (Figure 1F).

### Inhibitory effect of anti-miR-199a/-214 on CAFs and hPSC differentiation at gene level

**Figure 2:**
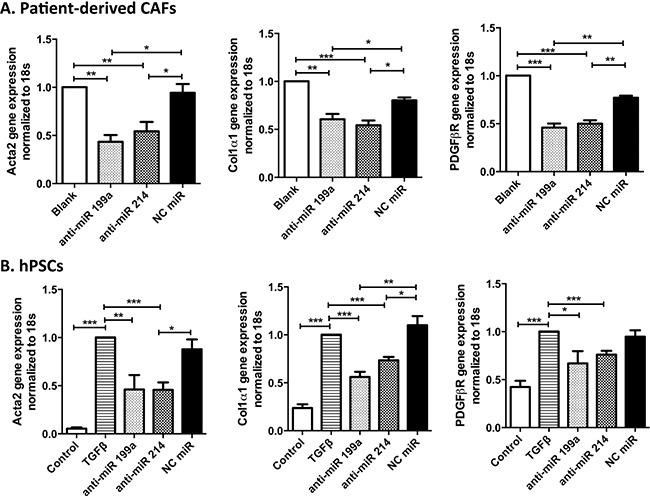
Effect of inhibition of miR-199a and -214 on CAFs and hPSCs transdifferentiation Transfection of anti-miR-199a or -214 hairpin inhibitors (50 nM) in CAFs, as CAFs were already activated in pancreatic tumor microenvironment (**A**), and TGFβ activated hPSCs (**B**) significantly inhibited the differentiation markers such as Acta2, Col-1a1, and PDGFβR at transcription level, as shown with qPCR. Data represent mean ± SEM for at least 3–4 independent experiments. *p < 0.05, **p < 0.01, ***p < 0.001.

To investigate whether inhibition of miR-199a or miR-214 dedifferentiates patient-derived CAFs and also hinders the differentiation of hPSCs into myofibroblasts, we transfected CAFs and hPSCs with their hairpin inhibitors and studied their effect at gene expression levels. Our results showed that both anti-miR-199a and -214 significantly reduced the expression of differentiation or myofibroblast markers such as Acta2, Col-1α1 and PDGFβR, at the transcriptional level in both CAFs and hPSCs (Figure 2A, 2B). These results indicate that both miR-199a and -214 are involved in differentiation of hPSCs into myofibroblasts.

### Inhibitory effect of anti-miR-199a/-214 on hPSC differentiation at protein level

**Figure 3:**
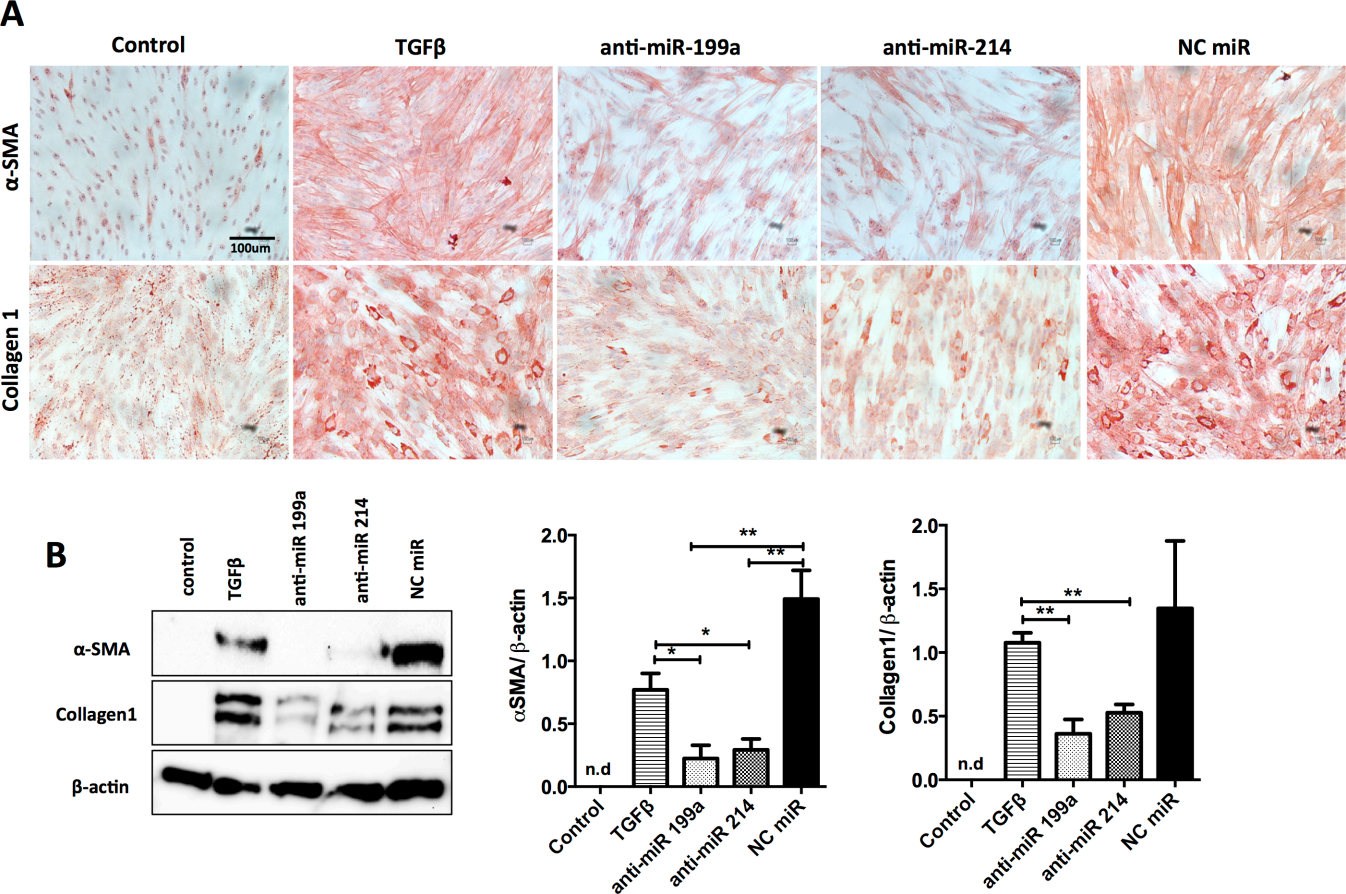
Effect of inhibition of miR-199a and -214 on hPSCs transdifferentiation Transfection of anti-miR-199a or -214 hairpin inhibitors (50 nM) in hPSCs significantly inhibited the TGF-β-induced differentiation markers such as αSMA and collagen-1 at protein levels, as shown with immunocytochemical stainings (**A**) and Western blot and densitometry analyses of the blots (**B**) compared to control (without TGFβ) and negative control miR. Data are presented as mean ± SEM, **p < 0.01, *p < 0.05, ***p < 0.001, *n* = 3. n.d. denotes non-detectable.

We further investigated the inhibitory effects of anti-miRs on the activation of hPSCs at the protein levels using immunocytochemical staining and Western Blot analyses. Both immunostaining and Western blot data clearly showed that anti-miR-199a and -214 significantly reduced TGF-β1-induced expression of myofibroblast phenotypic markers α-SMA and Collagen1 (Figure 3A, 3B). These results demonstrate that both miR-199a and miR-214 are involved in the differentiation of hPSCs into myofibroblasts.

### Effect of anti-miR-199a/-214 on the migration and proliferation of hPSCs

**Figure 4:**
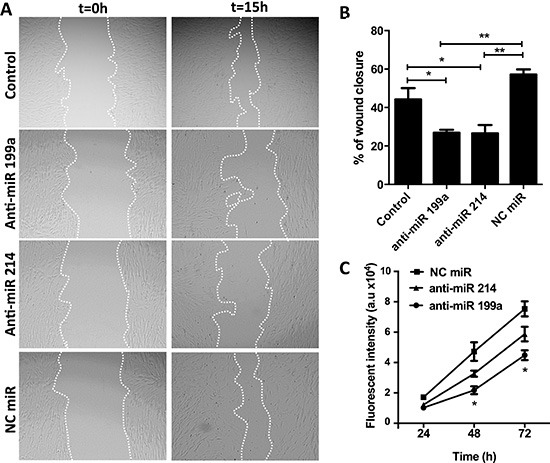
Effect of anti-miR-199a and -214 on migration and proliferation of hPSCs (**A**) Representative microscopic images (magnification, 40×) showing the effect of anti-miRs (50 nM) on the migration of hPSCs and (**B**) their semi-quantitative image analysis. (**C**) Cell growth curve is showing that transfection of hPSCs with anti-miR-199a (50 nM) significantly reduced the PSC proliferation compared to negative control anti-miR while anti-miR-214 showed only moderate inhibitory effects. A.u. denote arbitrary unit. Data are representative of three independent experiments. Mean ± SEM, *p < 0.05, **p < 0.01.

We investigated the effect of anti-miR-199a and -214 on migration and proliferation of hPSCs using scratch assay (wound healing assay) and Alamar Blue assay, respectively. Pretreatment of hPSCs with anti-miR-199a or -214 led to a significant inhibition of the closure of the wound (scratch gap) compared to the control cells. As shown in Figure 4A, control hPSCs and hPSCs transfected with control anti-miR (NC) rapidly migrated into the gap formed by the scratch made in the cell monolayer covering up to 45–50% of the gap within 15 h. In contrast, hPSCs transfected with anti-miRs (199a or 214) migrated at much slower rate, filling up only 25% of the gap (Figure 4B). Furthermore, we examined the effect of anti-miRs on the cell growth of the activated hPSCs for three days. We found that anti-miR-199a reduced the cell growth significantly whereas anti-miR-214 showed only moderate inhibitory effects (Figure 4C). These data demonstrate that both miR-199a and miR-214 are involved in regulation of hPSC migration while miR-199a is also involved in the proliferation of hPSCs.

### Effect of anti-miR-199a/-214 on the paracrine activity of hPSCs

**Figure 5:**
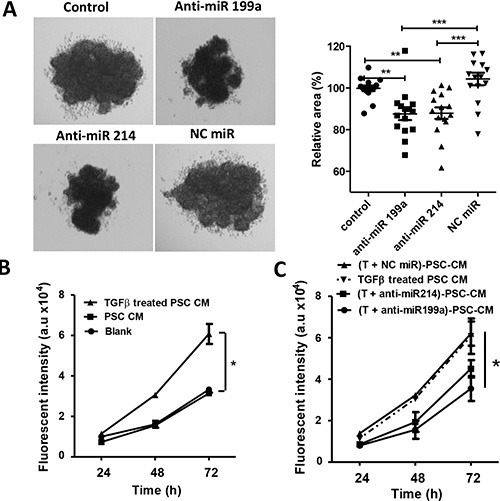
Effect of anti-miR-199a and -214 on the heterospheroid formation and hPSC-induced tumor cell proliferation (**A**) Heterospheroids were prepared by co-culturing Panc-1 tumor cells and hPSCs (control or transfected with anti-miRs) together using hanging drop method. In total, 15 drops were analyzed per condition, and the experiments were performed in three independent settings. (**B**) Panc-1 tumor cell growth curves after incubation with conditioned medium obtained from Panc-1 (self-control), control hPSCs, and TGFβ-activated hPSCs. (**C**) Panc-1 tumor growth curves after incubation with conditioned medium obtained from TGFβ-activated hPSCs transfected with anti-miR-199a (50 nM) significantly reduced the Panc-1 proliferation compared to negative control anti-miR. Cell growth was measured with AlamarBlue dye. A.u. denote arbitrary unit. Data are representative of three independent experiments. Data is represented as mean ± SEM. *p < 0.05, **p < 0.01, ***p < 0.001.

After studying the direct effect of anti-miRs on hPSCs, we further investigated the hPSC-induced paracrine effects on tumor cells and endothelial cells. To study the effect of miR-199a and -214 on hPSC-induced paracrine effects on tumor cells, we generated heterospheroids by co-culturing hPSCs (control or transfected with anti-miRs) together with Panc-1 tumor cells in 1:1 ratio using the hanging drop method. We found that spheroids composed of Panc-1 and hPSCs (transfected with anti-miR-199a and -214) formed smaller spheroids compared with control hPSCs (Figure 5A). Furthermore, we collected conditioned media from hPSCs with or without activation with TGFβ to collect PSC-secreted cytokines and growth factors. We found that addition of the conditioned media obtained from TGFβ-activated hPSCs to Panc-1 tumor cells induced their growth rate more than those treated with the conditioned media obtained from the non-activated hPSCs (Figure 5B). Interestingly, treatment with anti-miR-199a or -214 reduced PSC-induced proliferation of tumor cells compared with that of negative control miR treated hPSCs (Figure 5C).

### Effect of anti-miR-199a/214 on the hPSC-induced effect on endothelial cells

**Figure 6:**
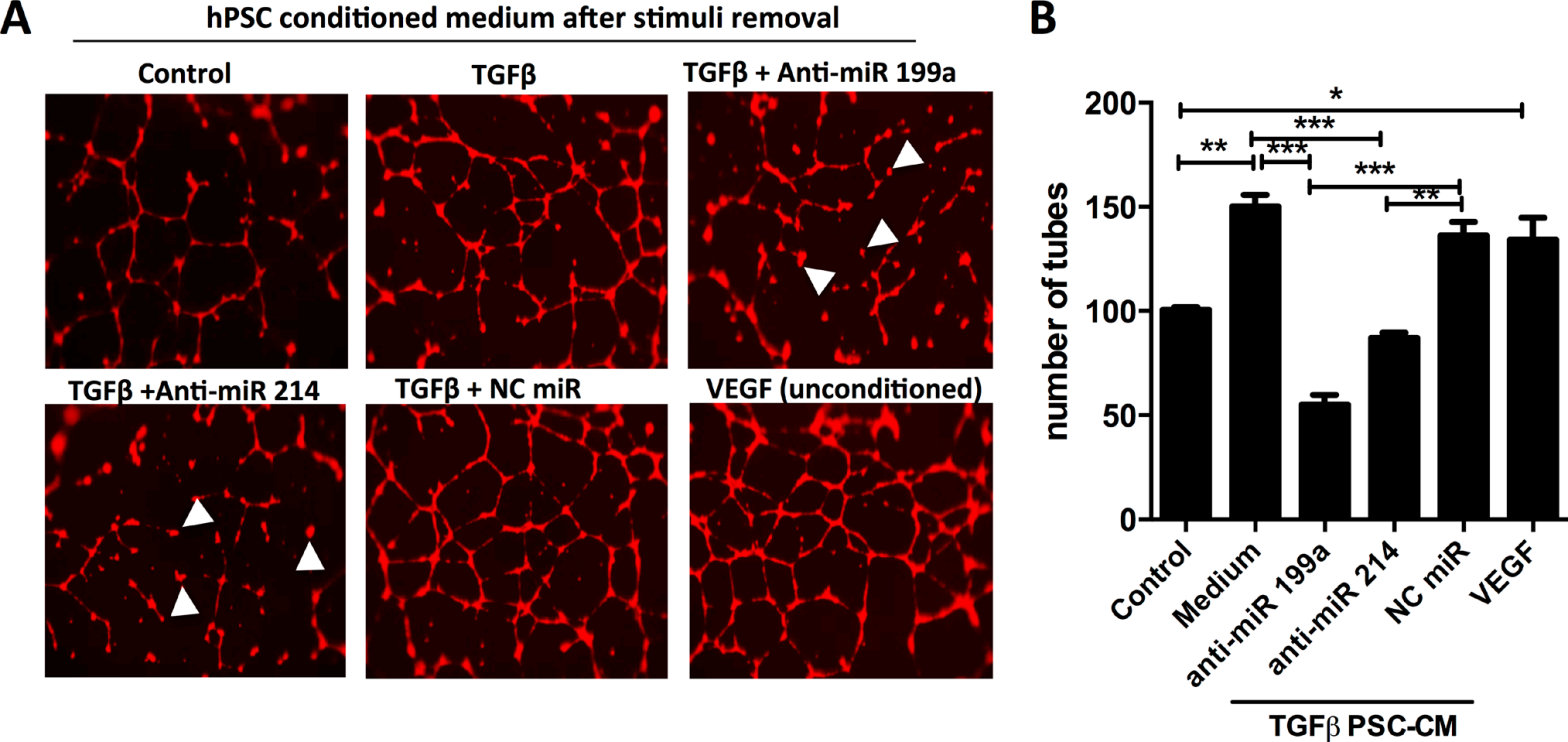
Effect of anti-miR-199a and -214 on hPSC-mediated paracrine effect on endothelial cells (**A**) Representative microscopic images (40× magnification) of human endothelial cell tube formation by HUVECs after incubation with conditioned media collected from control hPSCs or TGFβ-activated hPSCs with or without transfected with anti-miR-199a, anti-miR-214 or anti-miR negative control (NC). (**B**) Quantitative analysis of a number of tubes formed. VEGF (10 ng/ml) was used directly on HUVECs as a positive control. Mean ± SEM, *n* = 3, *p < 0.05, **p < 0.01, ***p < 0.001; unpaired student's *t*-test.

To study the effect of inhibition of miR-199a and -214 in hPSC for their effect on endothelial cells, we performed *in vitro* endothelial cell tube formation assay. When HUVECs were incubated with conditioned media derived from TGF-β-stimulated hPSCs, they significantly developed more capillary-like structures (so-called tubes) compared with those treated with unstimulated PSC-derived conditioned media (Figure 6A, 6B). The increase in the number of capillary tubes was similar to those with the treatment with human VEGF, an endogenous angiogenesis-inducing growth factor. Interestingly, the conditioned media derived from hPSCs transfected with anti-miR-199a and -214, yet activated with TGFβ, had a significantly diminished tube formation (indicated by arrowheads) compared to the conditioned media from TGF-β-stimulated hPSCs or negative control (control anti-miR plus TGFβ-stimulated hPSCs) (Figure 6A, 6B). Although we observe a much decrease in the tube formation with conditioned medium from anti-miR-199a treated PSCs, we observed no cell death in endothelial cells microscopically after staining with calcein-red. These data indicate that inhibition of miR-199a and -214 in PSCs inhibits TGF-β-activated PSC-induced endothelial cell activation.

### Identification of miR-199a/-214 candidate targets using bioinformatics tools

**Figure 7:**
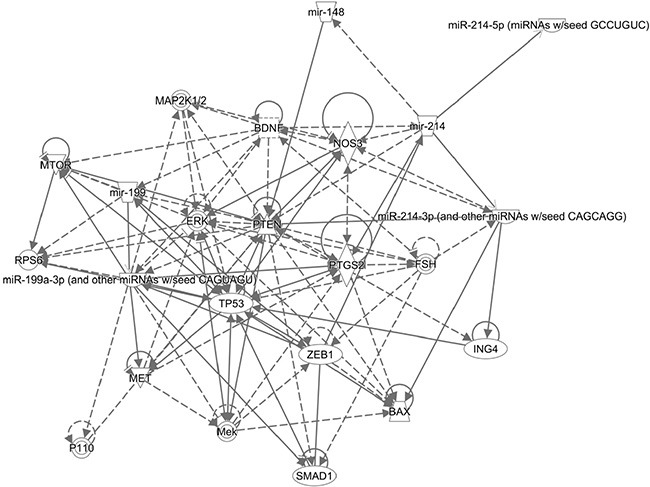
Network predicted by IPA for miR-199a and -214 target genes Ingenuity IPA pathway analysis predicted target genes for miR-199a and miR-214. Pointed arrowheads represent activating relationships while solid or dotted edges indicate direct or indirect relationships, respectively.

We explored the predicted genes that might be responsible for the multiple functions of miR-199a and miR-214. Ingenuity IPA revealed the most biological processes that might be responsible for cellular activation, differentiation, and proliferation (Figure 7). Interestingly, network generated by IPA revealed the interactions of miR-199a with some of the potential target genes such as mTOR, TP53, and SMAD1 and for miR-214 such as PTEN, BAX and ING4 (Table 1). On the other hand, miR database (microrna) revealed the common target genes (TMEM161B, QKI, COL4A5, KCNH8, CELSR2) for these two miRNAs based on the miR score.

**Table 1:** Direct relationship with miR-199a and -214 target genes

miRNA	Target genes	NCBI Reference Sequence	Functions related to CAFs
hsa-miR-199a-3p	mTOR	NM_004958.3	Proliferation, differentiation
	TP53	NM_000546.5	Differentiation, apoptosis, Self-renewal
	SMAD1	NM_005900.2	Differentiation
hsa-miR-214-3p	PTEN	NM_001304717.2	Proliferation, differentiation
	BAX	NM_001291428.1	Apoptosis
	ING4	NM_016162.3	Cell proliferation, apoptosis, senescence and invasion

## DISCUSSION

In the present study, we explored two miRNAs miR-199a-3p and miR-214-3p for their potential therapeutic role in the activation of pancreatic stellate cells (PSCs) and PSC-induced pro-tumorigenic effects in pancreatic cancer. Using *in-situ* hybridization technique, we confirmed that these miRs were overexpressed in pancreatic tumor stroma and subsequently their high expression was confirmed in patient-derived pancreatic CAFs and TGF-β-activated hPSCs. Inhibition of miR-199a or miR-214 using hairpin inhibitors led to the inhibition of TGFβ-induced hPSC activation, differentiation, and proliferation. Furthermore, we demonstrated that inactivation of hPSCs with miR inhibitors significantly reduced the hPSC-induced paracrine pro-tumorigenic effects on tumor cell proliferation and endothelial cells.

The role of stroma in pancreatic cancer remains contradictory and context dependent as summarized by Gore and Korc [35]. Depletion of stroma might not be the right strategy to show the significnace of CAFs in pancreatic stroma, as there might be “good (anti-tumoral)” and “bad (pro-tumoral)” CAFs [10]. Since there are no markers to distinguish these differential CAF phenotypes, normalization of CAFs, in general, might be a right strategy to inhibit their paracrine signals. Recently, Madsen *et al.* have demonstrated that reversal of CAFs to normal state using prolyl hydroxylase domain protein 2 inhibitor leads to reduction in CAF-induced metastasis in lungs and liver in a breast co-injection tumor model [36].

So far, small drug molecules have been explored as anti-CAF agents [7, 9, 37, 38], whereas miRNAs for inhibiting CAFs in the pancreatic tumor are rarely explored [39, 40]. Since miRNAs are strong regulators of cellular processes [25], we in the present study explored miRNA as novel therapeutic targets to inhibit the protumorigenic activities of hPSCs. We selected miR-199a-3p and miR-214-3p as main miRNAs to investigate their therapeutic potential in PSCs, as they were shown to be upregulated in activated rat PSCs, lung fibrosis and cardiac remodeling [27, 33, 41]. CAFs are mainly derived from the resident PSCs after their transdifferentiation into myofibroblasts [13]. In this study, we differentiated primary hPSCs into CAF phenotypic myofibroblastic cells using human TGFβ, as appeared from their stretched enlarged morphology and induced a-SMA gene (Acta2) and protein levels. Interestingly, induction of both miR-199a and miR-214 was also established in these cells, indicating a relationship of these miRNAs with PSC differentiation. Furthermore, inhibition of miR-199a or miR-214 using hairpin inhibitors dedifferentiated patient-derived CAFs, which was confirmed at gene expression levels. Interestingly, TGF-β−induced activation and differentiation as well as migration and cell growth of hPSCs was inhibited by anti-miR-199a or anti-miR-214, confirming the significance of these miRs in controlling PSCs’ phenotypic behavior.

Many studies have shown that the differentiated hPSCs elicit pro-tumorigenic effects by secreting growth factors and cytokines, and thereby induce tumor progression, invasion, and metastasis [11, 17]. Interestingly, in the present study we confirmed that activated hPSCs induced tumor cell growth as well as activation of human endothelial cells. Treatment of hPSCs with anti-miR-199a or anti-miR-214 abrogated hPSC-induced Panc-1 tumor cell growth, spheroid formation and inhibited endothelial cells activation. These data signify that inhibition of miR-199a and miR-214 in hPSCs leads to inhibition of PSC-induced pro-tumorigenic effects *in vitro*, representing them interesting miRNAs to develop for potential gene therapy.

Many potential direct and indirect target genes were predicted using IPA pathways. The key direct targets for miR-199a are mTOR, p53, Smad1, and miR-214 are PTEN, ING4, and Bax. As shown by many studies, PTEN and TP53 are frequently dysregulated in many human malignancies and play a crucial role in the regulation of proliferation and differentiation [42–44]. These studies have shown pro-tumoral effects of p53 inactivation in the stromal fibroblasts, as well as that genetic inactivation of PTEN in CAFs, potentiate both onset and progression of carcinomas [42–47]. It has been reported that BMP2 signaling activate Smad1, in turn inhibits TGF-β-induced PSC activation and ECM formation [48]. Moreover, Smad1 is shown to activate mTOR-induced protein synthesis [49], a critical molecule for inducing proliferation and differentiation in different cell types [50, 51]. BAX (Bcl-2-associated X protein) is a pro-apoptotic gene, and its alteration modulates fibroblasts function by disrupting apoptosis pathway [52]. Furthermore, BAX is also involved in p53-directed apoptosis [53]. ING4, an inhibitor of growth family, member 4, is a tumor suppressor protein that can interact with p53, inhibit cell growth, and induce apoptosis [54]. Additionally, these genes might also be dysregulated in pancreatic stroma that plays a crucial role in activation and differentiation.

In conclusion, this study unravels miR-199a-3p and miR-214-3p as novel therapeutic targets in pancreatic CAFs and hPSCs, as their inhibition led to dedifferentiation of pancreatic CAFs and inhibition of differentiation of hPSCs to myofibroblasts. This study also highlights the roles of these miRNAs in controlling myofibroblast phenotypic behavior such as differentiation, migration and proliferation that may lead to their future applications for fibrotic diseases. Nevertheless, silencing of miR-199a or miR-214 in hPSCs/CAFs may represent a novel therapeutic option for the development of novel therapies for this devastating disease.

## MATERIALS AND METHODS

### Materials

All miRIDIAN microRNA human hairpin inhibitors were purchased from Thermo Scientific, Germany. miScript inhibitor Negative control and HiperFect transfection reagent were purchased from Qiagen (Venlo, The Netherlands). AlamarBlue was purchased from Invitrogen (Breda, The Netherlands).

### Cells

Human pancreatic cancer associated fibroblasts (CAFs) were isolated from pancreatic tumor tissue obtained during pancreatic surgery from patients with resectable pancreatic head adenocarcinoma and cultured by the outgrowth method, as explained elsewhere [55]. Human primary pancreatic stellate cells (hPSCs) were obtained from ScieneCell (Carlsbad, CA) and were cultured in specified medium provided by the manufacturer, supplemented with penicillin/streptomycin. Cells were used less than the passage 9 and seeded on a Poly-L-Lysine-coated plate. Human umbilical vein endothelial cells (HUVEC), obtained from Lonza (Breda, The Netherlands), were cultured in EBM-2 medium supplemented with EGM-2 MV. Pancreatic cancer cell line (Panc-1) was obtained from American type culture collection (ATTC, Rockville, MD), were cultured in Dulbecco's modified Eagle's medium (DMEM, PAA, The Netherlands) supplemented with 10% FBS and antibiotics (50 U/ml penicillin and 50 ng/ml streptomycin).

### *In situ* hybridization (ISH) assay

MicroRNA *in situ* hybridization (ISH) was performed using locked nucleic acid (LNA) probes for miR-199a-3p and miR-214-3p together with a MicroRNA ISH kit for FFPE tissues (Exiqon). FFPE pancreatic tumor samples were obtained from Laboratory of pathology of East Netherlands, Hengelo and the ethical approval was obtained from the local human ethical committee. Analyses were performed according to manufacturer's protocol using the parameters stated below. Briefly, FFPE slides were deparaffinized, treated with proteinase-K (15 μg/mL) for 10 min and incubated with microRNA probe (50 nM) for one h at 56°C. For visualization, anti-DIG block solution with the alkaline phosphatase (AP) antibody (1:800) (Roche Diagnostics) supplemented with goat serum (Jackson Immunoresearch) and NBT-BCIP tablets (Roche Diagnostics) were used.

### Quantitative real-time PCR

hPSCs were seeded in 24 well plate (2 × 10^4^ cells/well) or 12 well plate (6 × 10^4^ cells/well) in complete medium. After 18 h, cells were transfected with anti-miR-199a and anti-miR-214 hairpin inhibitors for 24 h. Thereafter, cells were activated with TGF-β1 (5 ng/ml) for 24 h, then total RNA was isolated using the GenElute^™^ Mammalian Total RNA Miniprep Kit and the RNA amount was measured by a NanoDrop^®^ ND-1000 Spectrophotometer (Wilmington, DE). Subsequently, cDNA was synthesized with iScript^™^ cDNA Synthesis Kit (BioRad, Veenendaal, The Netherlands). 10ng cDNA was used for each PCR reaction. The real-time PCR primers (see Table 2) for human αSMA, Collagen1α1, PDGFβR, and RPS18 were purchased from Sigma (The Netherlands). Gene expression levels were normalized to the expression of the house-keeping gene 18s.

**Table 2:** Primers used for quantitative real-time PCR

Gene	Forward	Reverse
α-SMA	CCCCATCTATGAGGGCTATG	CAGTGGCCATCTCATTTTCA
Collagen1α1	GTACTGGATTGACCCCAACC	CGCCATACTCGAACTGGAAT
PDGFβR	AGGCAAGCTGGTCAAGATCT	GCTGTTGAAGATGCTCTCCG
RPS18	TGAGGTGGAACGTGTGATCA	CCTCTATGGGCCCGAATCTT

### Western blot analyses

hPSCs were seeded into a 12 well plate (6 × 10^4^ cells/well) in complete medium. After 18 h, cells were transfected with anti-miR-199a and anti-miR-214 hairpin inhibitors for 24 h. Then, cells were activated with TGF-β1 (5 ng/ml) and after 48 h cells were lysed with RIPA buffer (Thermo-Scientific) containing protease inhibitor cocktail. Cell lysis was centrifuged at 10,000 g for 10 min, and the supernatant fractions were collected for Western blot analysis. Equal amounts of proteins were loaded on 10% Tris-Glycine gel (Thermo Scientific) and transferred onto PVDF membranes (Thermo Scientific). The blots were probed with various primary antibodies αSMA, Collagen1 and β-actin at different dilutions (see Table 3) was incubated overnight at 4°C, followed by incubation at RT for 1 h with species specific horseradish peroxidase (HRP) conjugated secondary antibody. The proteins were detected by Pierce^™^ ECL Plus Western Blotting Substrate kit (Thermo Scientific) and exposed to FluorChem^™^ M System (ProteinSimple, CA). The protein levels were normalized with β-actin and quantified by Image J Software (NIH, MD).

**Table 3:** Primary and secondary antibodies used for the immune-blotting analysis

Antibody	Source	Dilution
Mouse monoclonal α-SMA	Sigma	1:500
Goat polyclonal Collagen1α1	Southern Biotech	1:250
Mouse monoclonal β-actin	Sigma	1:10000
HRP-conjugated rabbit anti-goat IgG	DAKO	1:2000
HRP-conjugated goat anti-mouse IgG	DAKO	1:2000

### Immunocytochemical staining

hPSCs were seeded in 24 well plates (2 × 10^4^ cells/well) in complete medium. After 18 h, cells were transfected with anti-miR-199a and anti-miR-214 hairpin inhibitors for 24 h. After that, cells were activated with TGF-β1 (5 ng/ml) for 48 h and cells were fixed and immunostained for αSMA, and Collagen1 as described elsewhere [56].

### Cell proliferation assay

hPSCs were transfected and activated with TGFβ1 as described above and after 24 h cells were washed and detached with trypsin. Then, cells were plated at a density of 5 × 10^3^ cells/well in 96 well plates. Cell growth was analyzed over a period of 3 days by adding 10 μl of AlamarBlue dye (Invitrogen) in 100 μl media to each well and the plates were incubated at 37°C. After four hours, the fluorescence reading (540 nm excitation and 590 nm emission wavelength) was recorded with VICTOR^™^ plate reader (PerkinElmer, Waltham, Massachusetts).

To study the indirect effect of hPSCs on tumor cells (Panc-1), hPSCs conditioned medium was collected. hPSCs were seeded into a 12-well plate (6 × 10^4^ cells/well) in complete medium. After 18 h, cells were incubated in serum-free media and transfected with anti-miR-199a and anti-miR-214 (50 nM). After 24 h, they were incubated with TGF-β1 (5 ng/ml) for another 24 h. Then, cells were washed 3 times and incubated with a fresh starved medium for 24 h. This collected conditioned medium was put on tumor cells for cell proliferation. Panc-1 cells were plated at a density of 2.5 × 10^3^ cells per well in 96-well plate. Cell growth was analyzed as stated above.

### Migration assay

hPSCs were seeded in a 24-well plate (4 × 10^4^ cells/well) for 18 h and transfected with anti-miR-199a and anti-miR-214 hairpin inhibitors and allowed them to become confluent. A standardized scratch was made using a 200 μl pipette tip fixed in a custom-made holder. Then, cells were washed and incubated with fresh serum-free media without growth factors. Images were captured at *t* = 0 h and *t* = 15 h, under an inverted microscope. Images were analyzed by Image J software to calculate the area of the scratch and represented as the percentage of wound closure compared to that of control (control hairpin inhibitors) cells.

### Spheroid formation assay

Spheroids containing a mixture of hPSCs and tumor cells were prepared using the hanging drop method with minor modifications as described elsewhere [57]. hPSCs transfected with anti-miRs are trypsinized and suspended in culture medium to a concentration of 3 × 10^5^ cells/ml. The hPSCs and Panc-1 cell suspensions were mixed with a ratio of 1:1. Approximately five drops (20 μl/drop containing 6 × 10^3^ cells) were dispensed onto a lid of a cell culture dish. Then, the lid was inverted and placed over cell culture dish containing PBS for humidity. The spheroids were grown for six days and imaged under an inverted microscope, and size (area) of the spheroid was measured digitally using ImageJ software.

### Tube formation assay

The PSC paracrine effects of miRNA inhibitors on endothelial cells (HUVEC) were examined using the matrigel tube formation assay. The PSC-conditioned medium was collected after different treatments as mentioned above and added to HUVECs (2 × 10^4^ cells/well) plated on the BD Biocoat angiogenesis 96-well plate (BD Biosciences, Bedford, MA) and the tube formation assay was performed. 96-well plate with pre-coated matrigel was incubated at 37^°^C for 30 minutes for polymerization. Subsequently, HUVECs were seeded into the plate containing the PSC-conditioned medium. As a positive control, VEGF (10 ng/ml, Peprotech) was added directly to HUVECs. After 24 h incubation, tubes per well were labeled with CellTrace^™^ Calcein Red-Orange AM (Invitrogen, Breda, The Netherlands) at 8 μg/ml concentration as per manufacturer's protocol. For quantification, tubes were analyzed to count the tube joints and represented as the number of tubes.

### Statistical analyses

All values are expressed as a mean ± standard error of mean (SEM). Statistical analysis of the results was performed either by a two-tailed unpaired student's *t*-test for comparison of two treatment groups or a one-way ANOVA to compare multiple treatment groups. Differences were considered significant minimally at *p* < 0.05.

## SUPPLEMENTARY MATERIALS FIGURE


