# Supplementary material for: MicroRNA-199a and -214 as potential therapeutic targets in pancreatic stellate cells in pancreatic tumor
Source: Oncotarget. 2016 Feb 24;7(13):16396–408. doi: 10.18632/oncotarget.7651 (PMC4941323; doi:10.18632/oncotarget.7651)
Supplement: Supplementary file 1 [file oncotarget-07-16396-s001.pdf]

MicroRNA-199a and -214 as potential therapeutic targets in pancreatic stellate cells in pancreatic tumor

Supplementary Materials

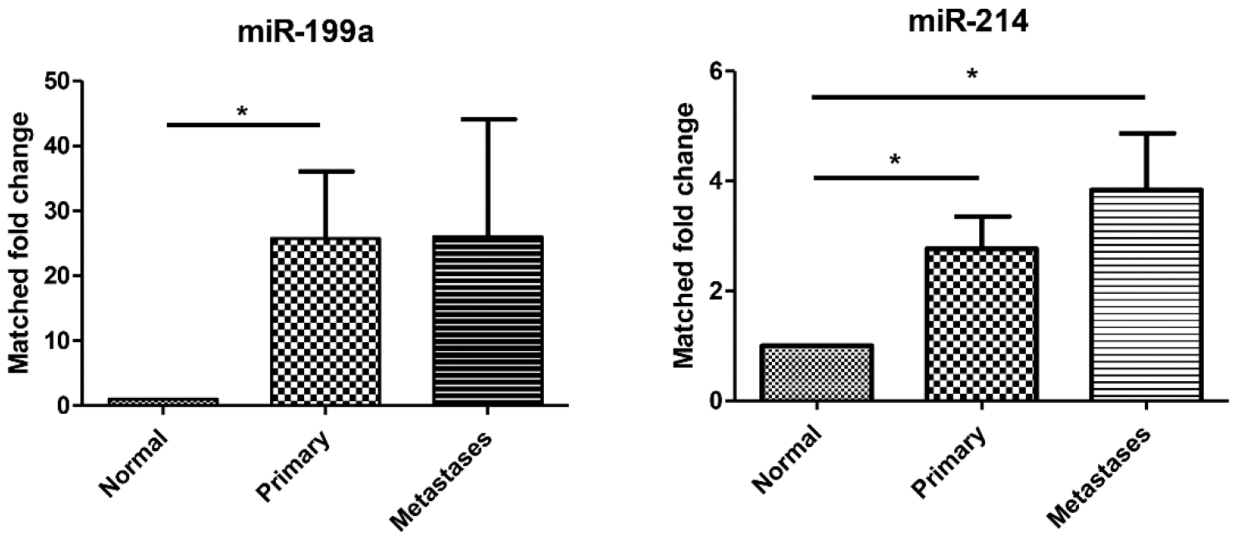

Supplementary Figure S1: Data showing miRNA-199a and -214 expression levels in matched colorectal carcinoma tumor and liver metastasis in patients. The stromal region from non-tumor (normal), malignant tumor at the primary tumor site and the liver metastatic site was isolated using laser captured microdissection and analyzed by RT-qPCR. Data represent $n = 8$ matched samples from patients. Mean + SEM, $*p < 0.05$.
